# Fatal coma in a young adult due to late-onset urea cycle deficiency presenting with a prolonged seizure: a case report

**DOI:** 10.1186/s13256-015-0741-2

**Published:** 2015-11-23

**Authors:** Majid Alameri, Mustafa Shakra, Taoufik Alsaadi

**Affiliations:** Department of Neurology, Sheikh Khalifa Medical City (SKMC), P.O. Box 51900, Abu Dhabi, United Arab Emirates

**Keywords:** Ornithine transcarbamylase deficiency (OTC), Hyperammonemia, Coma

## Abstract

**Introduction:**

Unexplained hyperammonemic coma in adults can be a medical dilemma in the absence of triggering factors and known comorbidities. Ornithine transcarbamylase deficiency presents most commonly with hyperammonemic coma. Although a rare disorder, ornithine transcarbamylase deficiency is the most common of the urea cycle disorders, which can occur both in children, and less commonly, in adults. The urea cycle disorder is usually acquired as an X-linked trait, and very rarely, similar to our reported case, may be acquired as a “new” mutation. Mutations that lead to later-onset presentations may lead to life-threatening disease and may be unrecognized, particularly when the first clinical symptoms occur in adulthood.

**Case presentation:**

We report the case of a previously healthy 17-year-old white man who developed a prolonged seizure and a rapid decline in mental status leading to coma over a 3-day period. Analysis of the *OTC* gene showed a 119G variant, which was identified in exon 2 of the *OTC* gene by sequencing.

**Conclusions:**

A diagnosis of ornithine transcarbamylase deficiency should be considered in adult patients who present with unexplained hyperammonemic coma and for all adult patients presenting with cryptogenic new-onset seizure and laboratory finding of elevated blood ammonia levels. This reported case highlights the importance of early recognition of this potentially reversible cause of life-threatening encephalopathy, as timely recognition and appropriate treatment can be lifesaving.

## Introduction

Ornithine transcarbamylase (OTC) deficiency is a urea cycle X-linked defect, which is considered the most prevalent inherited defect of the urea cycle [1]. While typical presentation occurs in infancy, a few reported cases of late-onset presentations in adults are well described [2]. However, the delayed presentation is most commonly seen in partial OTC deficiency [2]. Males are usually more commonly, and most severely, affected than females [3]. The presentation varies, according to the degree of X-chromosome inactivation, from mild symptoms of fatigue and lethargy, to severe encephalopathy, and coma leading to death [4].

The urea cycle is the primary metabolic pathway for the excretion of nitrogenous wastes such as urea. OTC catalyzes the mitochondrial reaction of ornithine (the end product of the extraction of urea from arginine) with carbamoyl phosphate (the first storage form of ammonia) to produce citrulline. Deficiency of OTC leads to the formation of excess carbamoyl phosphate; some of which is excreted as an orotic acid. When this pathway is overwhelmed, hyperammonemia results. Thus, patients with OTC deficiency tend to have hyperammonemia, elevated levels of orotic acid in the urine, and low plasma citrulline [5].

Males and heterozygous females with partial OTC deficiency can present from infancy to adulthood. One observational study of 21 male patients found the age at presentation ranges from 2 months to 44 years [6]. Furthermore, it was observed that male patients who were older at presentation had a diverse form of presenting symptoms and were associated with higher mortality rates [6]. This data illustrates the phenotypic variability of OTC deficiency even in the mild form of the disease (partial OTC deficiency), where a hyperammonemic crisis can be precipitated easily, progressing very rapidly into a life-threatening condition. For all individuals with OTC deficiency, a wide spectrum of neuropsychological complications have been described, including developmental delay, intellectual disability, attention deficit hyperactivity disorder (ADHD), and executive function deficits [7].

Here, we report the case of a previously healthy 17-year-old man who developed intermittent nausea and vomiting for 1 week, followed by a witnessed new-onset prolonged generalized tonic-clonic seizure, and a rapidly deepening coma over a 3-day period. A week prior to presentation, he had started to take a high-protein supplement. Investigations for liver disease, drug and alcohol use, and infections were all negative. His blood NH3 concentration was remarkably high on initial presentation, but started to decrease with medical therapy and dialysis. During recovery, our patient developed ventilator-associated pneumonia treated with antibiotics, but unfortunately the course was further complicated by severe *Clostridium difficile* colitis and miliaria profunda, which progressed rapidly resulting in our patient’s death. The diagnosis of OTC deficiency was confirmed by genetic analysis showing a 119G variant, which was identified in exon 2 of the *OTC* gene by sequencing. This case illustrates the importance of recognizing this very unusual presentation of new-onset seizure and with laboratory finding of elevated blood ammonia levels related to underlying partial OTC deficiency. Timely recognition and appropriate treatment for these cases can be lifesaving.

## Case presentation

A 17-year-old white man, previously healthy, was admitted following a reported new-onset generalized tonic-clonic seizure. The seizure was witnessed by his mother. She reported a continuous convulsion lasting for almost 6 minutes associated with urinary incontinence. Prior to admission, he had generalized body fatigue, intermittent nausea and vomiting for 1 week, without any associated features such as fever, chills, diarrhea, abdominal pain, chest pain, shortness of breath, and weight loss. He was diagnosed as gastritis in a primary health care clinic, and was treated symptomatically. Upon further inquiry, he reported recently joining a local fitness club and starting on high-protein supplement for 1 week prior to the development of the presenting symptoms.

**Fig. 1:**
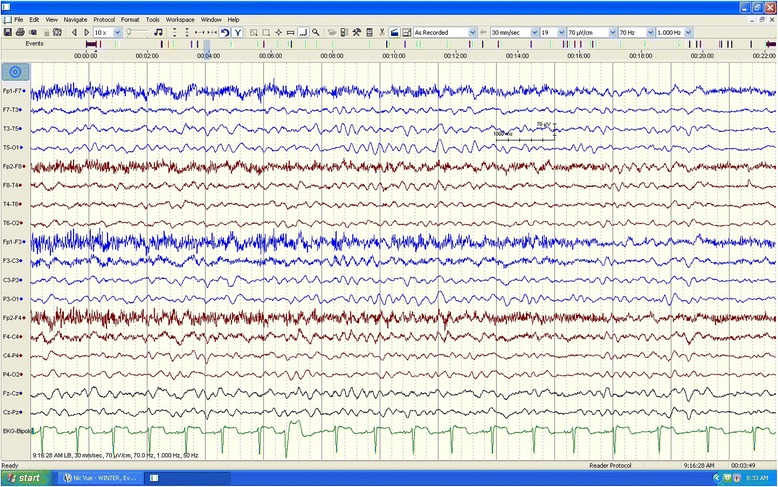
This electroencephalography portion of long-term tracing demonstrates background slowing in the range of 6–7 HZ. There is no evidence of consistent focal or lateralizing findings nor was there any evidence of clear epileptiform discharge. High-pass filter is set at 1 Hz; low-pass filter at 70Hz; notch filter is off

On admission, he was afebrile with normal vital signs. The neurologic examination revealed that he was intermittently aroused to painful stimuli. His Glasgow Coma Scale score was 10 (E4, V2, M4), with the remainder of the examination being nonfocal. Initial laboratory data showed a normal leukocyte count 10.7 × 10^9^/L (N 4.5–13 × 10^9^/L), normal glucose, electrolytes and calcium levels. His liver function tests were as follows: alkaline phosphatase: 179 IU/L (N 40–129 IU/L), AST = 40 IU/L (*N* <40 IU/L), ALT = 166 IU/L (<41 IU/L), total bilirubin = 8 microm/L (≤17 microm/L), an ammonia level of 787 micromol/L (N 16–60 umol/L) with a normal coagulation panel. His initial chest X-ray, computed tomography (CT) scan of the brain, and magnetic resonance imaging/magnetic resonance angiography (MRI/MRA) of the brain were all normal. His initial prolonged electroencephalography (EEG) showed mild diffuse background slowing without any evidence of electrographical seizures, or clear epileptiform discharges (Fig. 1).

**Fig. 2:**
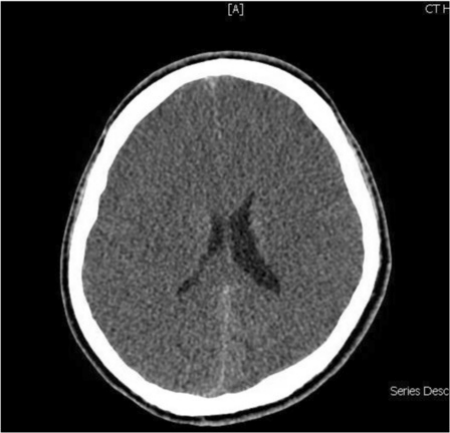
Computed tomography brain scan without contrast showing a generalized loss of the supratentorial grey-white matter differentiation with effacement of sulci indicating increasing degree of diffuse cerebral edema. Asymmetry of lateral ventricles is seen with slit-like appearance of anterior horns. An effacement of the basal cisterns can be noted as well

**Fig. 3:**
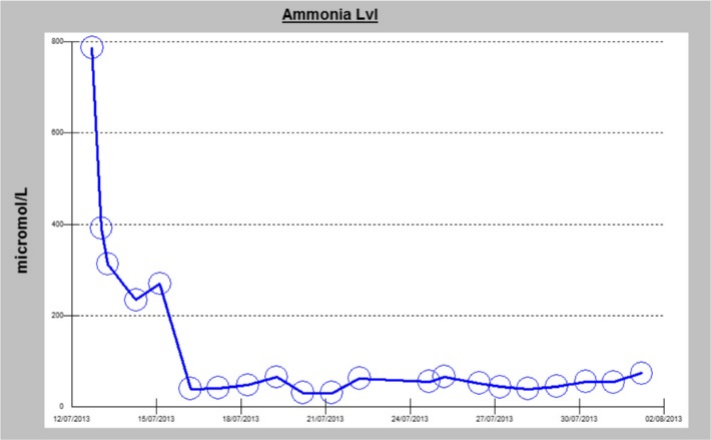
Diagram showing a timeline of the measured ammonia level in the serum, observation of an exceptionally elevated level of 787 umol/L (normal range = 11–35 umol/L) can be noted on the initial presentation, responding to medical therapy with a gradual decline during the hospital stay (ammonia scavenger therapy arginine, sodium benzoate and intermittent hemodialysis)

An ultrasound scan of his abdomen and pelvis with liver Doppler showed normal hepatic parenchyma and patent hepatic vessels without any other abnormality. Over the first 24 hours of admission, he became less responsive and his ammonia level failed to normalize despite lactulose therapy. He was intubated for airway protection. A repeat CT of the brain was done on the second day of critical care admission, showing diffuse effacement of sulcal spaces with decreased attenuation of the cerebral parenchyma, suggesting mild diffuse cerebral edema (Fig. 2). In view of his persistently elevated serum ammonia levels and brain edema, dialysis was initiated, and plasma and urine amino acid analysis and urine organic acid quantitation were performed. The blood test showed low level of citrulline (7 μM; normal 19–62 μM), while the urine test revealed elevated levels of orotic acid (27.7 mmol/mol creatinine; normal 0–1.3 mmol/mol creatinine). Based on clinical and laboratory findings, urea cycle disorder was strongly suspected and our patient was started on arginine and an ammonia scavenger therapy, sodium benzoate, with intermittent hemodialysis. After the third hemodialysis session along with the medical therapy, our patient’s ammonia levels had significantly decreased over the course of treatment (median 55, average 184 μmol/L, range 30–787 μmol/L) (Fig. 3), but without any observable improvement in his level of consciousness.

**Fig. 4:**
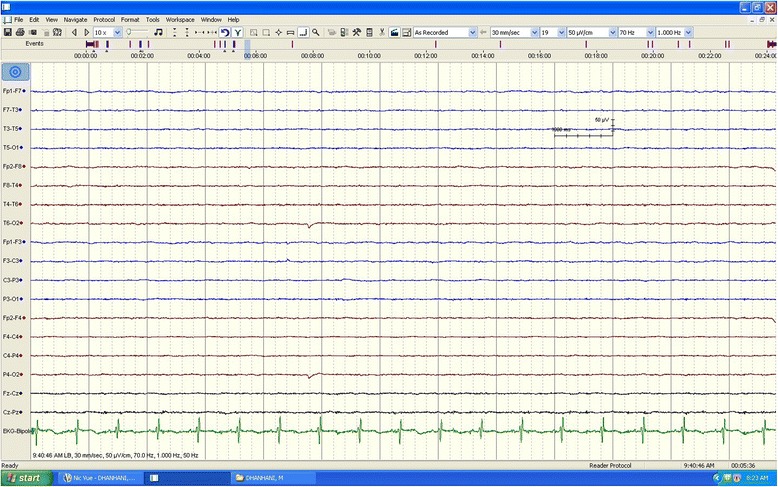
At a sensitivity of 50 μV/CM, a repeated electroencephalogram obtained several days after admission shows diffusely attenuated and poorly organized background activity. This electroencephalography pattern is suggestive of profound generalized disturbance of cerebral activity. The calibration mark represents 1 second and 50 μV

At that time, a repeat EEG showed a very severely attenuated, poorly organized, and nonreactive EEG that was observed at high sensitivity, indicating a profound generalized disturbance of cerebral activity (Fig. 4). He was kept on maintenance medical therapy and intermittent hemodialysis. His ammonia level was subsequently maintained within normal range without improvement in the level of consciousness, failing multiple ventilation weaning trials. During the recovery period, he developed ventilator-associated pneumonia treated with intravenous piperacillin/tazobactam for 10 days, but unfortunately, the course was further complicated by severe *Clostridium difficile* colitis, and miliaria profunda that ended with cardiopulmonary arrest and death despite aggressive restitution.

The initial genetic testing for the OTC deficiency did not show any deletion and no duplication was identified in the *OTC* gene by multiplex ligation-dependent probe amplification (MLPA) analysis. However, and because of the strong clinical suspicion, this result was not considered as conclusive, as it could not exclude OTC variants, where any variants outside of the analyzed region might not be detectable by MLPA and still might be present. Sequence analysis of the *OTC* gene was recommended and the diagnosis was confirmed showing 119G variant, which was identified in exon 2 of the *OTC* gene. Based on these results, all of our patient’s siblings were recommended to be screened and were referred for genetic consulting.

## Discussion

Adult-onset OTC deficiency is a rare cause of encephalopathy. The persistent elevated ammonia level may be the first clue to the diagnosis. Most of the adult-onset patients remain asymptomatic, till they present with a rapid decline in mental status, either spontaneously, or following a heavy protein challenge, as was the case in our patient [8]. Our case, similar to the previously few reported cases of OTC deficiency, presented with a rapidly worsening coma that, unfortunately, can be potentially fatal [9]. Indeed, death is an usual outcome of the disease in its mild forms. However in severe cases, death may be an evitable outcome, either because of liver failure or, similar to our patient, due to intercurrent severe illness and fatal inflammatory response [10]. On the other hand, seizures have been reported rarely as the first manifestation of OTC deficiency. Our patient had a reported prolonged seizure, unlike generalized convulsive seizures, which typically last less than 2 minutes. We realize the limitation of depending on witnesses to estimate the duration of a convulsive seizure; however, due to the concern of missing a nonconvulsive seizure following the reported prolonged seizure, a 6-hour continuous EEG was obtained, ruling out that possibility.

The basis of treatment of the acute status of hyperammonic coma is to target the plasma ammonia level to ≤200 μmol through the use of ammonia scavenger treatment to allow excretion of excess nitrogen, which is expected to reduce the risk of neurologic damage, and if needed, a renal replacement therapy can further aid to lower the ammonia levels. Our patient’s ammonia level responded well to treatment, but his mental status did not. This lack of correlation between declining ammonia levels and improvement in mental status is quite puzzling. In fact, a recently reported series of five cases of adult-onset OTC deficiency have demonstrated similar findings, where three of their patients had a normal ammonia level days after initiation of treatment but their cognitive status had not improved, leading to death [11].

Liver transplantation is typically considered in those who have frequent hyperammonic episodes [12]. After the acute phase, routine measurement of plasma ammonia, amino acids, and liver function every 3 to 6 months may be required [7].

The diagnosis of OTC deficiency in adults may be difficult, as clinical manifestations are nonspecific, and often episodic in nature, which add more difficulties in considering the diagnosis. In fact, early diagnosis and prevention of the metabolic decompensations in adult-onset cases is crucial to avoid this, unfortunately fatal, and other negative cognitive outcomes [13]. Moreover, laboratory findings can be normal outside the acute phase. In all cases, molecular analysis is the method of choice to confirm the diagnosis of OTC deficiency. Indeed, a recently published study analyzed the clinical, biochemical and genetic parameters of 90 patients from a single center and revealed various molecular and genetic mutations in patients with OTC deficiency across different age groups.

## Conclusions

Urea cycle disorders are one of the complex medical metabolic emergencies that occur in infancy until adulthood and must be treated promptly to avoid severe brain injury and death. However, there is a very limited number of case reports in the literature for cases presenting in young adults. Our case should call on all physicians to consider this diagnosis for any adult presenting with unexplained hyperammonic coma or unexplained change in mental status. In these cases, every effort should be made to screen for this condition very rapidly and to offer immediate treatment along with precise genetic counseling. Future efforts should look into widening the knowledge about the combined effect of genetic factors (that is mutations in the *OTC* gene, variants in modifier genes or epigenetic features) and environmental conditions to determine the phenotypic expression of OTC deficiency.

## Consent

Written informed consent was obtained from the patient's legal guardian for publication of this case report and any accompanying images. A copy of the written consent is available for review by the Editor-in-Chief of this journal.
